# Expanding the molecular toolbox for *Lactococcus lactis*: construction of an inducible thioredoxin gene fusion expression system

**DOI:** 10.1186/1475-2859-10-66

**Published:** 2011-08-09

**Authors:** François P Douillard, Mary O'Connell-Motherway, Christian Cambillau, Douwe van Sinderen

**Affiliations:** 1 Department of Microbiology, University College Cork, Cork, Ireland; 2 Alimentary Pharmabiotic Centre, University College Cork, Cork, Ireland; 3 Architecture et Fonction des Macromolécules Biologiques, UMR 6098 Centre National de la Recherche Scientifique and Universités d'Aix-Marseille I & II, Campus de Luminy, Case 932, 13288 Marseille Cedex 09, France; 4 Department of Veterinary Sciences, University of Helsinki, Agnes Sjöbergin katu 2, 00790 Helsinki, Finland

**Keywords:** nisin, thioredoxin, expression system, *Lactococcus lactis*

## Abstract

**Background:**

The development of the Nisin Inducible Controlled Expression (NICE) system in the food-grade bacterium *Lactococcus lactis *subsp. *cremoris *represents a cornerstone in the use of Gram-positive bacterial expression systems for biotechnological purposes. However, proteins that are subjected to such over-expression in *L. lactis *may suffer from improper folding, inclusion body formation and/or protein degradation, thereby significantly reducing the yield of soluble target protein. Although such drawbacks are not specific to *L. lactis*, no molecular tools have been developed to prevent or circumvent these recurrent problems of protein expression in *L. lactis*.

**Results:**

Mimicking thioredoxin gene fusion systems available for *E. coli*, two nisin-inducible expression vectors were constructed to over-produce various proteins in *L. lactis *as thioredoxin fusion proteins. In this study, we demonstrate that our novel *L. lactis *fusion partner expression vectors allow high-level expression of soluble heterologous proteins Tuc2009 ORF40, Bbr_0140 and Tuc2009 BppU/BppL that were previously insoluble or not expressed using existing *L. lactis *expression vectors. Over-expressed proteins were subsequently purified by Ni-TED affinity chromatography. Intact heterologous proteins were detected by immunoblotting analyses. We also show that the thioredoxin moiety of the purified fusion protein was specifically and efficiently cleaved off by enterokinase treatment.

**Conclusions:**

This study is the first description of a thioredoxin gene fusion expression system, purposely developed to circumvent problems associated with protein over-expression in *L. lactis*. It was shown to prevent protein insolubility and degradation, allowing sufficient production of soluble proteins for further structural and functional characterization.

## Background

The food-grade bacterium *L. lactis *subsp. *cremoris *in conjunction with the Nisin Inducible Controlled Expression (NICE) system [1-3] has been extensively used over the last few decades as a valuable bacterial expression system for large-scale production of homologous or heterologous proteins [4], metabolic studies [5], or membrane proteins [6]. The NICE system is based on the well characterized nisin-dependent, quorum-sensing mechanism of *L. lactis *[2,3,7]. It was initially exploited in *L. lactis *for heterologous protein overexpression and subsequently implemented in several other Gram-positive bacteria [2,3,7-10]. Typically, the genetically-engineered strain *L. lactis *subsp. *cremoris *NZ9000 is employed as expression host, as its chromosome contains the signal transduction genes *nisR *and *nisK *involved in the nisin-induced transcriptional control of the P*nisA *promoter [3]. Any genes cloned downstream this nisin-inducible promoter P*nisA *can be expressed in a controlled manner upon addition of nisin to the bacterial culture [3]. However, production of recombinant proteins can be problematic in *L. lactis*, as over-expressed proteins may be subject to poor expression, stability and/or solubility. Such drawbacks are intrinsically associated with the prokaryotic cell machinery limitations and therefore are inherent to all bacterial expression systems, representing a significant bottleneck in high level production of soluble proteins.

In *E. coli*, a 'microbial cell factory' of choice for producing heterologous proteins [11,12], the development of the gene fusion technology proved to circumvent such recurrent and fundamental protein expression problems [13]. This technology involves the linkage of the protein of interest with a carrier protein to generate a fusion protein. Addressing solutions to problematic protein expressions, many fusion expression systems have been engineered and successfully employed, using solubility-enhancing fusion partners such as *Schistosoma japonicum *glutathione-S-transferase (GST) [14], *E. coli *maltose binding proteins (MBP) [15], *Staphylococcus *protein A [16], *E. coli *N-utilization substance (NusA) [17] and *E. coli *thioredoxin (TrxA) [18,19]. Along with the increasing number of fusion partners used, additional features have been successfully implemented to this technology, thus facilitating protein tagging, purification techniques and tag-mediated proteolytic cleavage [13,20,21]. The gene fusion technology provides a substantial palette of applications through the constant expansion of fusion gene expression systems available in *E. coli*. Nevertheless, the adaptation of these existing fusion partner systems to other expression hosts is sparse, even though significant progress has been made to develop new molecular tools and methods in alternative prokaryotic and eukaryotic expression systems [1,22,23]. The expression host *L. lactis *is currently lacking such a solubility-enhancing expression system to improve its spectrum of biotechnological applications, as *L. lactis *featured a number of benefits over other expression bacterial hosts, *e.g*. being a food-grade expression host, and the absence of endotoxins, extracellular proteinases and spores.

As part of our study on the structure-function analysis of lactococcal phage-host recognition and penetration, we attempted to over-express a number of proteins encoded by the lactococcal phage Tuc2009 in *L. lactis*. However, initial expression studies of individual protein subunits of Tuc2009 phage revealed such proteins often suffer from degradation, poor expression or result in insoluble protein aggregates, also called inclusion bodies (data not shown). The development of a fusion-based gene expression system in *L. lactis *could provide a novel strategy to express soluble proteins and avoid the use of laborious and spurious renaturation procedures. Among the numerous fusion partners employed, LaVallie *et al*. described the construction of an *E. coli *thioredoxin (TrxA) gene fusion system [19]. In most cases, *E. coli *thioredoxin fusion proteins were soluble, correctly folded and biologically active [19]. The *E. coli *thioredoxin thus appears to represent a good candidate for an *L. lactis *fusion-based gene expression system: small size of the fusion partner (11.67 kDa), ability to accumulate in a soluble form at high levels in the cytoplasm, steric accessibility of N- and C-termini of TrxA for protein fusions [19] and efficient generic protein purification methods available, *i.e*. immunoprecipitation or affinity chromatography [13,24].

In the present study, we report on the construction of two new *L. lactis *thioredoxin-fusion gene expression vectors harbouring the nisin-controlled expression (NICE) system. We evaluated the efficiency of the newly-constructed fusion gene expression system, by producing individual proteins or protein complexes that initially could not be expressed or were not soluble in *L. lactis*. Our data indicate that the *L. lactis *thioredoxin-fusion vectors represent a very valuable addition to the *L. lactis *genetic toolbox, in particular for the over-production of soluble proteins.

## Methods

### Bacterial strains, media, growth conditions and nisin preparation

Bacterial strains described in this study are listed in Table 1. *L. lactis *subsp. *cremoris *NZ9000 [3] and NZ9700 [3,6,7] were cultured at 30°C under static conditions in GM17 broth (M17 broth [25]; Oxoid, UK) with 0.5% (w/v) D-glucose (Sigma)). *L. lactis *transformants were selected on GM17 agar plates supplemented with 5 μg/ml chloramphenicol (Sigma). The supernatant from overnight cultures of the nisin-producing strain *L. lactis *NZ9700 was filter-sterilized and used in this study as a source of the inducer nisin [6].

**Table 1:** Bacterial strains and plasmids used in this study

Strains or plasmids	Relevant characteristics	Reference or source
*Strains*		
*L. lactis*		
NZ9000	MG1363 containing *nisRK *genes,	[3]
NZ9700	expression host of the NICE system Nisin producing *L. lactis *strain	[3,7]
*Plasmids*		
pNZ8048	Standard *L. lactis *expression vector, Cm^r^	[3]
pTX8048	pNZ8048 derivative harbouring the TrxA system, contains a His-tag cloned in frame	This study
pTX8049	pNZ8048 derivative harbouring the TrxA system	This study
pNZ8048-UAL	pNZ8048 encoding Tuc2009 *bppU, bppA, bppL *as an operon	This study
pTX8048-UAL	pTX8048 encoding Tuc2009 *bppU, bppA, bppL *as an operon	This study
pNZ8048-40	pNZ8048 encoding Tuc2009 *orf40*	This study
pTX8048-40	pTX8048 encoding Tuc2009 *orf40*	This study
pNZ8048-0140N	pNZ8048 encoding N-terminal His-tagged Bbr_0140	This study
pNZ8048-0140C	pNZ8048 encoding C-terminal His-tagged Bbr_0140	This study
pTX8048-0140	pTX8048 encoding Bbr_0140	This study

Cm, chloramphenicol.

### DNA amplification and cloning

Oligonucleotide primers were purchased from Eurofins MWG GmbH (Germany) and are listed in Table 2. Genomic DNA from Tuc2009 or *B. breve *UCC2003 was extracted as previously described [26,27]. Plasmids and primers are listed in Tables 1 and 2, respectively. High-fidelity hot start KOD DNA polymerase (Novagen, UK), restriction enzymes (Roche GmbH, Germany) and T4 DNA ligase (Promega, USA) were used as recommended by the relevant manufacturers. Plasmid DNA was electroporated into *L. lactis *NZ9000 as described by Holo *et al*. [28].

**Table 2:** Oligonucleotide primers used in this study

Primer	Sequence (5'-3')	Comments
pTX48-F	AGCCCATGGGCGATAAAATTATTCACCTGACT	Forward primer of *trxA*
pTX48-R	AGCCTGCAGGATCC*CTTGTCGTCGTCGTC*ACCAGAAGA**ATGATGATGATGATGGTG**CATATGGCCAGAAC	Reverse primer of *trxA *flanked by an enterokinase cleavage site and a His-tag
pTX49-R	AGCCTGCAGGATCCCATATGGCCAGAACCAGAAC	Reverse primer of *trxA*
UAL-F	AGCAGCCATGGCAGAACATTTTATAAC	Forward primer of *bppU *for pNZ8048
UAL-R	AGCAGCACTAGTTTA**GTGATGGTGATGGTGATG**ATTCCGATAAAGTTTTACAATC	Reverse primer of *bppL *for pNZ8048
UALX-F	AGCAGCGGATCCATGACAGAACATTTTATAAC	Forward primer of *bppU *for pTX8048
UALX-R	AGCAGCACTAGTTTAATTCCGATAAAGTTTTACAATC	Reverse primer of *bppL *for pTX8048
orf40-F	AGCAGCCCATGGGGCGGCTACTAAGTCGCCACTTGCATAAAT	Forward primer of *orf40 *for pNZ8048
orf40-R	AGCAGCACTAGTTTA**GTGATGGTGATGGTGATG**TAAGTGATAGCCATAAGCAA	Reverse primer of *orf40 *for pNZ8048
orf40X-F	AGCAGCGGATCCATGGGGCGGCTACTAAGTCGCCACTTGCATAAAT	Forward primer of *orf40 *for pTX8048
orf40X-R	AGCAGCACTAGTTTATAAGTGATAGCCATAAGCAA	Reverse primer of *orf40 *for pTX8048
0140N-F	TGCATCCCATGGAT**CATCACCATCACCATCACCATCAC**CATCACAGCCGGATTCTCAAGGAC	Forward primer of *bbr_0140 *for pNZ8048
0140N-R	TGCGCATCTAGATTATGCGATGTAGCTTTC	Reverse primer of *orf40 *for pNZ8048
0140C-F	AATTAACCATGGGCCGGATTCTCAAGGACAAGC	Forward primer of *bbr_0140 *for pNZ8048
0140C-R	TGCCGTTCTAGATTA**GTGATGGTGATGGTGATGGTG**ATGGTGATGTGCGATGTAGCTTTCGATGTGTAG	Reverse primer of *bbr_0140 *for pNZ8048
0140X- F	GACAAGGGATCCATGAGCCGGATTCTCAAG	Forward primer of *bbr_0140 *for pTX8048
0140X-R	AGCTCTCTAGATTATGCGATGTAGCTTTC	Reverse primer of *bbr_0140 *for pTX8048

In italic font, are indicated the sequencer encoding enterokinase cleavage site. In bold font, are indicated the polyhistidine tag-encoding sequence. The restriction sites are underlined.

### Construction of thioredoxin gene fusion expression vectors

**Figure 1:**
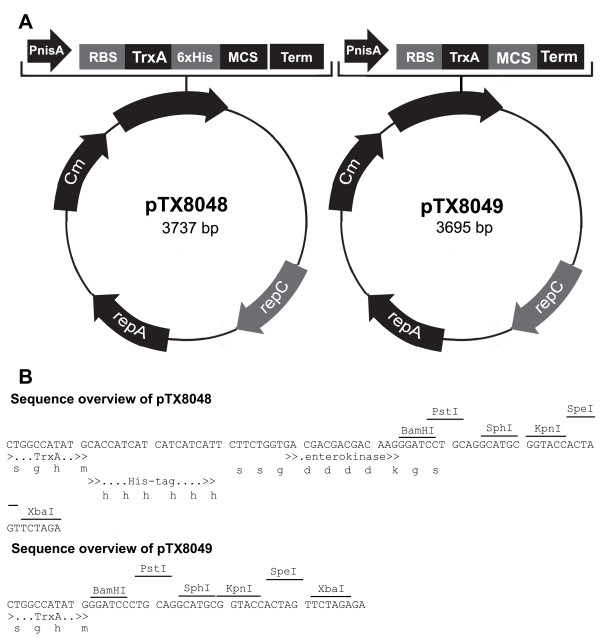
**Maps and sequence overview of pTX8048 and pTX8049**. (A) Vector maps of pTX8048 and pTX8049, which were constructed by insertion of the *E. coli *thioredoxin gene *trxA*, an enterokinase-specific cleavage site and a hexahistidine-tag as illustrated; (B) DNA sequence overview of the multiple cloning sites of pTX8048 and pTX8049. P*nisA*, nisin-inducible promoter; RBS, ribosome binding site; *trxA, E. coli *thioredoxin A-encoding gene; MCS, multiple cloning site; term, transcriptional terminator; Cm, chloramphenicol cassette. All restriction sites shown here are unique and can be used as cloning sites. The restriction site BamHI in pTX8048 and pTX8049 allows the in-frame cloning of the gene of interest.

The two expression vectors constructed in this study, called pTX8048 and pTX8049, are derived from the high-copy number vector pNZ8048 harbouring the NICE system and a chloramphenicol resistance marker [3]. The high-copy number expression vector pTX8048 was constructed as follows. The DNA fragment containing the *E. coli trxA *gene was amplified from the Gateway^® ^plasmid pETG-20a and flanked with a poly-histidine tag and an enterokinase cleavage site by PCR using primers pTX48-F and pTX48-R (Table 2). The resulting DNA amplicon was double-digested by NcoI and PstI, and then ligated to the similarly digested pNZ8048 plasmid (Figure 1). The ligation product was transformed into *L. lactis *NZ9000 and screened by colony PCR, and verified by restriction and sequencing analyses. The plasmid pTX8049 was constructed following a similar cloning strategy using primers pTX48-F and pTX49-R (Figure 1 and Table 2).

### Construction of expression vectors encoding thioredoxin fusion proteins

The gene encoding the Tuc2009 phage protein Tuc2009 ORF40 [29] was amplified from Tuc2009 DNA and flanked with a C-terminal hexa-histidine tag using primers orf40-F and orf40-R. The PCR product was digested with NcoI and SpeI and then ligated to pNZ8048 cut with *NcoI *and *SpeI*. The ligation product was transformed by electroporation into NZ9000 and screened by colony PCR, prior to restriction and sequencing analyses. Similarly, Tuc2009 *orf40 *was amplified using orf40X-F and orf40X-R, and cloned into the BamHI and SpeI sites of pTX8048. The restriction site BamHI in pTX8048 but also pTX8049 allows the in-frame cloning of a gene of interest, as shown in Figure 1. The three genes encoding the components of the Tuc2009 phage baseplate, i.e. *bbpU, bppA *and *bppL*, were amplified from Tuc2009 DNA and flanked with a C-terminal hexa-histidine tag using primers UAL-F and UAL-R. The PCR product was digested with NcoI and SpeI, and cloned into pNZ8048 cut with NcoI and SpeI. Similarly, the DNA region encompassing *bppU, bppA *and *bppL *was amplified using UALX-F and UALX-R, and then cloned into the BamHI and SpeI sites of pTX8048. The gene encoding the Bbr_0140 gene product was amplified from *Bifidobacterium breve *UCC2003 genomic DNA [30] and flanked with either a C- or an N-terminal hexa-histidine-encoding tag using, respectively, primer combinations 0140C-F and 0140C-R, or 0140N-F and 0140N-R. The PCR product was digested with NcoI and XbaI, and cloned into pNZ8048 cut with NcoI and XbaI. Similarly, *Bbr_0140 *was amplified using 0140X-F and 0140X-R, and subsequently cloned into BamHI and XbaI-restricted pTX8048.

### Protein expression assay

*L. lactis *NZ9000 cells harbouring one of the various plasmid constructs described in the Methods section were propagated overnight at 30°C in M17 broth [25] containing 0.5% (w/v) D-glucose and supplemented with 5 μg/ml chloramphenicol. Fresh GM17 media supplemented with 5 μg/ml chloramphenicol was inoculated with a 1/50 (v/v) overnight liquid culture and incubated at 30°C. When the optical density at 600 nm reached 0.4, protein expression was induced by the addition of nisin to a final concentration of 0.2% (v/v) [31]. Liquid culture was further incubated at 30°C for 4 hours and bacterial cells were harvested by centrifugation (3000 × g for 20 min at 4°C). Bacterial cell pellets were washed in 50 mM NaH_2_PO_4_, 300 mM NaCl, 10 mM imidazole, pH 8.0 and stored at -80°C until further use [31].

### Fractionation, SDS-PAGE, immunoblotting analysis and protein assays

Protein samples were prepared as described by Bahey-El-Din *et al*. [31]. Bacterial pellets were resuspended in 50 mM NaH_2_PO_4_, 300 mM NaCl, 10 mM imidazole, pH 8.0 supplemented with 30 mg/ml lysozyme and incubated for 30 min on ice. Cell preparations were then sonicated (8 × 10 sec with 10 sec on ice between each cycle) at maximum amplitude (MSE Soniprep 150, Sanyo). Insoluble and soluble fractions were separated by centrifugation at 14, 000 × g for 10 min at 4°C and stored at -20°C for further analysis. Sodium dodecyl sulfate-polyacrylamide gel electrophoresis (SDS-PAGE) was performed as previously described [32]. Proteins from 12.5% acrylamide gels were then transferred onto a PVD membrane (Millipore, UK) by electroblotting [33]. Mouse polyclonal antibodies directed against the poly-histidine tag or rabbit polyclonal antibodies directed against BppU, BppA and BppL were used as primary antibody [26]. Monoclonal anti-mouse or anti-rabbit antibodies coupled to horseradish-peroxidase (Sigma, USA) were used as secondary antibody. The membrane was developed using hydrogen peroxide and 4-chloro-1-naphthol (Sigma). His-tagged proteins were purified using the PrepEase^® ^Histidine-tagged Protein Purification kit (USB, OH, USA). Protein content was measured using the Bio-Rad Protein Assay (Germany), based on the Bradford protein quantification method.

### Enzymatic cleavage of TrxA fusion proteins using enterokinase

The fusion protein TrxA-Bbr_0140 was expressed and His-tagged purified as described above. Purified enterokinase from calf intestine (Roche GmbH, Germany) was used to cleave TrxA-Bbr_0140 according to manufacturer's instructions. TrxA-Bbr_0140 was dialyzed against 50 mM Tris buffer, pH 8.0, since phosphate buffer is known to significantly reduce enterokinase activity as indicated in the user's manual. Typically, 25 μg TrxA-Bbr_0140 was incubated with 0.6 μg enterokinase for 16 h at 20°C or 37°C (enterokinase: TrxA-Bbr_0140 ratio = 1:42). We tested the effect of SDS on enterokinase cleavage efficiency as recommended in the manufacturer's manual, by supplementing the reaction mixture with 0.1% (w/v) SDS. An equal volume of 2X SDS-PAGE loading buffer was added and the samples were boiled at 95°C for 5 min to inactivate enterokinase. The cleaved protein products were analyzed by SDS-PAGE.

## Results and discussion

### Description of the two *L. lactis *thioredoxin-fusion expression vectors

The two *L. lactis *thioredoxin-fusion expression vectors, called pTX8048 and pTX8049 (Figure 1) were employed in an attempt to express a number of proteins encoded by the lactococcal phage Tuc2009 and *B. breve *UCC2003. The anticipated translational fusions in both plasmids are placed under the transcriptional control of the nisin-inducible promoter P*nisA*, ensuring a tight control of protein expression in *L. lactis *[3]. The original ribosome binding site present in plasmid pNZ8048 was retained to ensure efficient translation of the fusion protein (Figure 1) as it had previously been reported that low expression of proteins may be due to inefficient translational initiation of mRNA [34]. The *E. coli *thioredoxin *trxA *represents the N-terminal portion of the fusion protein (Figure 1), promoting an efficient initiation of translation as previously described [13]. In addition, plasmid pTX8048 has been designed to join the thioredoxin C-terminus to the recombinant protein N-terminus with an amino-acid linker (SSGDDDDKGS) adapted from LaVallie *et al*. [19], consisting of serine (S), glycine (G), aspartic acid (D) and lysine (K) residues and a highly-specific enterokinase cleavage site (DDDDK) previously used in an *E. coli *thioredoxin fusion system [19] (Figure 1). The (SSG)X_5_(GS) residues act as flexible joints within the fusion protein connecting the thioredoxin C-terminus to the recombinant protein N-terminus. It facilitates access to the enterokinase cleavage site (DDDDK), to facilitate release of the mature protein [35]. In pTX8048, the thioredoxin-specifying sequence was modified to include a C-terminal hexa-histidine encoding tag, also termed 'Histidine-patch thioredoxin' in the originally developed *E. coli *fusion expression systems [36,37] enabling the purification of the fusion protein by Ni-TED affinity chromatography. The thioredoxin and the hexa histidine-tag are located upstream of the enterokinase cleavage site and can therefore be removed from the protein of interest (Figure 1). In comparison, pTX8049 only contains the *E. coli *thioredoxin gene *trxA *followed by a multiple cloning site to insert a gene of interest in an in-frame manner (Figure 1). The lack of additional purification tags or linkers in pTX8049 allows a greater level of flexibility in designing and constructing original fusion proteins, *i.e*. addition, choice and location of purification tags [13], peptide linkers [38] and specific cleavage sites (tobacco etch virus protease cleavage site, thrombin or factor Xa) [35] (Figure 1). Methods and performances to over-produce proteins using pTX8049 are identical to pTX8048, as they both share the same pNZ8048 backbone and range of bacterial expression hosts.

### Production of Tuc2009 ORF40 as a fusion protein

**Figure 2:**
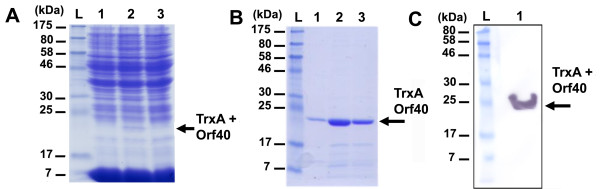
**Production of a small phage protein encoded by Tuc2009 ORF40**. (A) Protein gel analysis of soluble fractions of NZ9000 + pTX8048-40 without nisin induction (lane 1), and after induction with 0.2% (v/v) nisin (lane 2), soluble fraction of NZ9000 + pNZ8048-40 after 0.2% nisin induction (lane 3). (B) Protein gel of purified Tuc2009 ORF40 from NZ9000 + pTX8048-40. Lanes: L, prestained marker; 1-3, elution fractions. (C) Immunoblotting analysis showing Tuc2009 ORF40 using mouse anti-polyhistidine antibody as primary antibody. The Tuc2009 ORF40 protein product is indicated by an arrow.

In *E. coli *but also in *L. lactis*, the production of small proteins or peptides is often problematic, as proteins can be subject to degradation or can aggregate into inclusion bodies. Tuc2009 ORF40 is a small protein (7.65 kDa) with no known function and encoded by the lactococcal phage Tuc2009. We initially attempted to express the C-terminal His-tagged Tuc2009 ORF40 using the vector pNZ8048 in which ORF40 was cloned. The size of Tuc2009 ORF40 does not allow its detection by SDS-PAGE as the corresponding band would have been masked by the large amount of lyzozyme (14 kDa) required to lyse *L. lactis *NZ9000 (Figure 2, panel A). However, further immunoblotting analysis and Ni-TED affinity chromatography indicated that Tuc2009 ORF40 was not expressed in either soluble or insoluble form in NZ9000 (data not shown). We constructed the vector pTX8048-40 to express the fusion protein TrxA-Tuc2009 ORF40. Expression assays of TrxA-ORF40 in NZ9000 are shown in Figure 2. A distinct band corresponding to TrxA-ORF40 (21.8 kDa) was observed in the soluble fraction (Figure 2, panel A). Subsequently, TrxA-ORF40 was successfully purified by Ni-TED chromatography - availing of the peptide linker of pTX8048 containing a His-tag patch - and the total soluble fraction was analyzed by immunoblotting using anti-poly histidine antibodies (Figure 2, panels B and C). Using the thioredoxin fusion gene expression vector pTX8048, similar results were obtained for other small phage proteins, such as Tuc2009 ORF41 (12.8 kDa) and Tuc2009 ORF43 (11.9 kDa), where initial expression attempts using the original pNZ8048 NICE vector had also failed (data not shown). These results clearly demonstrate that small proteins when fused to the *E. coli *thioredoxin can be efficiently expressed in *L. lactis*.

### Production and purification of the Tuc2009 phage baseplate in *L. lactis *NZ9000

**Figure 3:**
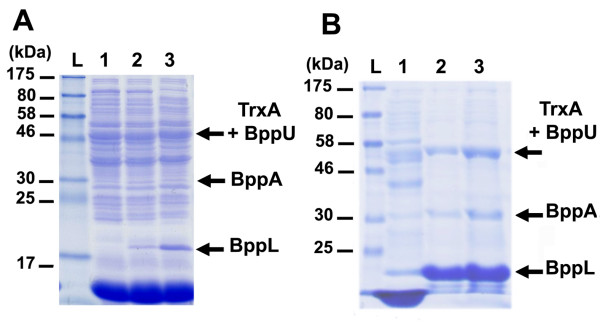
**Co-production of the Tuc2009 baseplate components BppU, BppA and BppL**. (A) Protein gel analysis of soluble fractions of NZ9000 + pTX8048-UAL without nisin induction (lane1), NZ9000 + pNZ8048-UAL following induction with 0.2% (v/v) nisin (lane 2) and NZ9000 + pTX8048-UAL following induction with 0.2% (v/v) nisin (lane 3). (B) Protein gel of Ni-affinity purified baseplate complex from NZ9000 + pTX8048-UAL soluble fraction. Lanes: L, prestained marker; 1, flow-through; 2-3, elution fractions.

**Figure 4:**
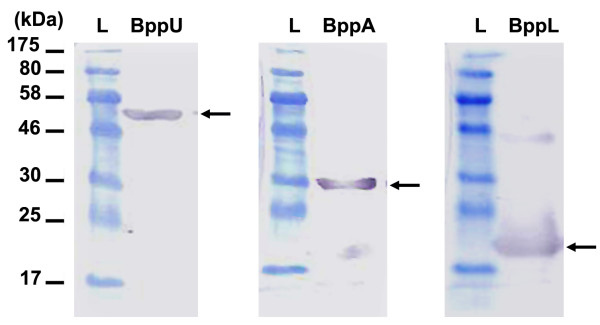
**Immunoblotting analysis of Tuc2009 baseplate components BppU, BppA and BppL**. Lanes: L, prestained marker. Immunoblotting analysis showing BppU, BppA and BppL using, respectively, anti-BppU, anti-BppA and anti-BppL rabbit polyclonal antibody as primary antibody. BppU, BppA and BppL are indicated by arrows.

Recombinant proteins may be improperly folded, preventing interactions with their protein partner(s). Such problems hamper further characterization of large hetero(multi)meric protein complexes, as certain protein-protein interactions may be prevented. The baseplate (Bpp) of Tuc2009 is a large multimeric protein complex involved in host recognition [39-43]. It consists of three proteins: the upper baseplate (BppU), the associated baseplate (BppA) and the lower baseplate (BppL) [26,41]. Although a low-resolution model of the Bpp complex has been proposed [41], the fine details of its intimate structure are not yet fully understood. Further functional and structural analyses of Bpp would be greatly facilitated if the Bpp complex could be over-expressed. Initial attempts to over-express the Bpp complex in *L. lactis *using the vector pNZ8048 in which the corresponding genes of the Bpp complex had been cloned demonstrated that BppU, BppA and BppL were produced, although at low levels. However, subsequent purification attempts did not allow the co-purification of the three Bpp complex components, *i.e*. BppU, BppA and BppL (data not shown). In Tuc2009 and also the closely related lactococcal phage TP901-1, the baseplate complex is associated with the initiation complex and the baseplate component BppU has been reported to be particularly vulnerable to degradation [41]. It was hoped that production of the Bpp complex, where BppU is fused to thioredoxin, would stabilize the Bpp complex, and that the presence of the His-tag in the peptide linker would facilitate co-purification of the heteromeric Bpp complex. In order to test this idea, plasmid pTX8048-UAL was generated and tested for Bpp complex expression and purification. Following induction, and cell lysis, total soluble protein fraction was resolved by SDS-PAGE (Figure 3). Although only one band corresponding to BppL (18.8 kDa) was visually detected in this way, further purification and immunoblotting analyses indicated that TrxA-BppU (50.4 kDa) and BppA (31.85 kDa) and BppL were all expressed and could be co-purified as a complex by affinity chromatography availing of the hexahistidine-tag of TrxA-BppU (Figures 3 and 4). Immunoblotting analysis was performed to check the integrity of the over-expressed proteins (Figure 4), which indicated the apparent absence of any degradation and/or sub-products. This result clearly demonstrated that the *L. lactis *Trx-fusion expression system is also suitable for the production and purification of intact heteromultimeric protein complexes.

### Over production of *Bifidobacterium breve *UCC2003 Bbr_0140 using pTX8048

**Figure 5:**
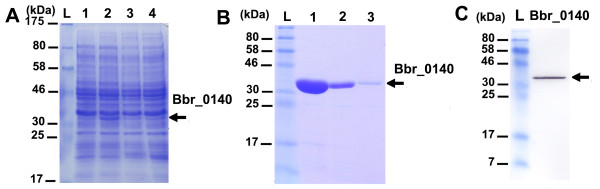
**Production of *B. breve *protein Bbr_0140**. (A) SDS-PAGE analysis of soluble fractions of NZ9000 + pTX8048-0140 without nisin induction (lane1), and following induction with 0.2% (v/v) nisin (lane 2), soluble fraction of NZ9000 + pNZ8048-0140C following 0.2% nisin induction (lane 3) and soluble fraction of NZ9000 + pNZ8048-0140N following 0.2% nisin induction (lane 4). (B) SDS-PAGE analysis of purified Bb_0140 from cell extracts of NZ9000 + pTX8048-0140 following 0.2% nisin induction. Lanes: L, prestained marker; 1-3, elution fractions. (C) Immunoblotting analysis showing Bbr_0140 using mouse anti-polyhistidine antibody as primary antibody. Bbr_0140 is indicated by an arrow.

In an effort to demonstrate the versatility of our thioredoxin system, we also attempted to over-produce a protein encoded by a bacterium that is unrelated to *L. lactis*. Bbr_0140 specifies a 200 amino-acid protein (23.5 kDa) encoded by the *Bifidobacterium breve *UCC2003 genome [30]. We initially attempted to express either C- or N-terminally His-tagged Bbr_0140 using the standard expression vector pNZ8048 in which we had cloned the coding sequence of Bbr_0140. However, using this expression system no Bbr_0140 protein product was detected by SDS-PAGE (Figure 5, panel A). We therefore constructed pTX8048-0140 to express the fusion protein TrxA-Bbr_0140 as described in Methods. Expression assays of TrxA-ORF40 in NZ9000 are shown in Figure 5. A distinct band corresponding to TrxA-Bbr_0140 (37.5 kDa) was observed in the soluble fraction, clearly demonstrating that TrxA-Bbr_0140 was successfully over-produced, while further analysis showed that this protein could be purified by Ni-TED affinity chromatography and visualized by immunoblotting using anti-poly histidine antibodies as a primary antibody (Figure 5, panels B and C). These results show that the Trx-fusion expression system for *L. lactis *is also suitable to produce heterologous proteins from a completely unrelated bacterial origin.

### Enterokinase cleavage of the thioredoxin fusion protein TrxA-Bbr_0140

**Figure 6:**
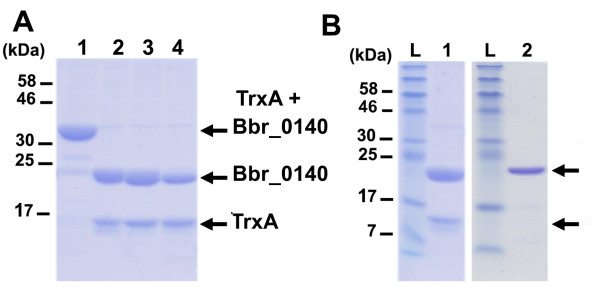
**Cleavage of the thioredoxin-Bbr_0140 fusion protein by enterokinase**. (A) Protein gel analysis of purified TrxA-Bbr_0140 incubated for 16 h at 20°C without enterokinase (lane 1), incubated for 16 h at 37°C with enterokinase (lane 2), incubated for 16 h at 37°C with enterokinase and 1% (v/v) SDS (lane 3), incubated for 16 h at 20°C with enterokinase and 1% (v/v) SDS (lane 4). (B) Protein gel analysis of purified Bbr_0140 after cleavage (lane1) and after cleavage and subsequent purification (lane 2).

The presence of a cleavage site is an important feature of this Trx-fusion expression system, as it allows the cleavage and release of the thioredoxin moiety from its fused protein of interest. Our vector pTX8048 possesses a peptide linker containing an enterokinase cleavage site (DDDDK) that connects the thioredoxin to the C-terminus of the fused protein. To test whether we could remove the Trx-moiety, purified thioredoxin-Bbr_0140 fusion protein was incubated with calf intestine enterokinase as described in the Methods section. As shown in Figure 6 (panel A), the thioredoxin-Bbr_0140 fusion protein was efficiently and specifically cleaved, as two products of 23.5 kDa and 14 kDa corresponding to the mature Bbr_0140 protein and the thioredoxin- linker product, respectively, were clearly observed by SDS-PAGE. The supplementation of the cleavage mixture with 0.1% (w/v) SDS did not improve the cleavage efficiency (Figure 6, panel A), indicating that the enterokinase cleavage site is equally accessible and cleavable in native and denaturing conditions. Further applications will dictate what cleavage conditions can be used. Under native conditions, subsequent purification of the cleaved Bbr_0140 protein was performed using Ni-TED chromatography. His-tagged thioredoxin-linker and uncleaved fusion proteins were retained on the nickel resins, whereas cleaved mature proteins were collected in the flow-through (Figure 6, panel B). However, the purified samples will still contain the enterokinase contaminant, although its corresponding band can not be observed on the protein gel (Figure 6, panel B). Additional/alternative chromatography techniques may be considered, such as ion exchange chromatography or gel filtration to further purify the mature protein following thioredoxin cleavage by removing the enterokinase contaminant [19]. Alternatively, the use of commercially available his-tagged enterokinase could also be considered and implemented to the purification procedure described in this study. Using the Bio Rad Protein Quantification Assay, we measured an average yield of 1.2 mg per litre of culture of purified Bbr_0140. It is noteworthy that protein yields are protein-dependent and not every protein will be expressed at the same level using pTX8048 and pTX8049. Mierau and colleagues previously reported that the original NICE system allows the production of very high levels of proteins, *e.g*. up to 300 mg of lysostaphin per liter of culture on a industrial scale [1]. Although the yields of production shown are significantly lower using our thioredoxin gene fusion systems, it is still a valuable tool as it allows the expression of soluble proteins that could not be expressed with the original NICE system. Also, the adjustment of key protein-specific parameters, such as medium composition and fermentation conditions could significantly improve the production yield of the target protein. The design and incorporation of an enterokinase cleavage site in pTX8048 is shown to be functional and further purification allows the final production of native and soluble heterologous proteins in *L. lactis*.

## Conclusions

The thioredoxin gene fusion system represents an attractive system to over-produce and purify proteins in *L. lactis *that exhibit poor or no expression, or produce insoluble proteins using conventional expression vectors. In this study, we have described the construction of an *L. lactis *Trx-fusion expression system and demonstrated its applicability by over-producing and purifying various proteins or complexes as soluble thioredoxin fusions. The benefits of the original *E. coli *thioredoxin fusion expression system have previously been demonstrated [18,19], and in this report we have shown that these are also applicable to the expression host *L. lactis*, when combined with the NICE system. This expression and purification tool offers a wide spectrum of applications in *L. lactis *and also other Gram-positive bacteria that can accommodate the NICE system, such as *L. plantarum *[44]. Although our study does not show the functionality of the overexpressed proteins, we are confident that the majority of such proteins are biologically active as based on numerous peer-reviewed studies using the original NICE system, as reviewed by Mierau *et al*. [1]. The protein production levels obtained in *L. lactis *using the thioredoxin fusion gene expression system allow further structural and biochemical analysis, such as X-ray crystallography analysis, antibody production, protein-protein interaction assays, and enzymatic assays.

## Competing interests

The authors declare that they have no competing interests.

## Authors' contributions

FPD performed all experiments described in this study, designed the different expression vectors and drafted the manuscript, except MOCM who performed cloning and protein production of Bbr_0140 proteins. CC participated in its design and its coordination. FDP and DVS conceived of the study, and participated in its design and coordination and wrote the manuscript. All authors read, edited and approved the final manuscript.

## References

[B1] Mierau I Kleerebezem M 10 years of the nisin-controlled gene expression system (NICE) in *Lactococcus lactis* Applied Microbiology and Biotechnology 2005 68 6 705 717 10.1007/s00253-005-0107-6 16088349

[B2] Kuipers OP Beerthuyzen MM de Ruyter PGGA Luesink EJ de Vos WM Autoregulation of nisin biosynthesis in *Lactococcus lactis *by signal transduction Journal of Biological Chemistry 1995 270 45 27299 27304 10.1074/jbc.270.45.27299 7592991

[B3] Kuipers OP de Ruyter PGGA Kleerebezem M de Vos WM Quorum sensing-controlled gene expression in lactic acid bacteria Journal of Biotechnology 1998 64 1 15 21 10.1016/S0168-1656(98)00100-X

[B4] Rigoulay C Poquet I Madsen SM Gruss A Expression of the *Staphylococcus aureus *surface proteins HtrA1 and HtrA2 in *Lactococcus lactis* FEMS Microbiology Letters 2004 237 2 279 288 15321674 10.1016/j.femsle.2004.06.046

[B5] Burgess C O'Connell-Motherway M Sybesma W Hugenholtz J van Sinderen D Riboflavin production in *Lactococcus lactis*: potential for *in situ *production of vitamin-enriched foods Applied and Environmental Microbiology 2004 70 10 5769 5777 10.1128/AEM.70.10.5769-5777.2004 15466513 PMC522069

[B6] Kunji ERS Slotboom D-J Poolman B *Lactococcus lactis *as host for overproduction of functional membrane proteins Biochimica et Biophysica Acta (BBA) - Biomembranes 2003 1610 1 97 108 10.1016/S0005-2736(02)00712-5 12586384

[B7] Kuipers OP Beerthuyzen MM Siezen RJ de Vos WM Characterization of the nisin gene cluster *nisABTCIPR *of *Lactococcus lactis* European Journal of Biochemistry 1993 216 1 281 291 10.1111/j.1432-1033.1993.tb18143.x 7689965

[B8] Hasper HE de Kruijff B Breukink E Assembly and stability of nisin Lipid II Pores Biochemistry 2004 43 36 11567 11575 10.1021/bi049476b 15350143

[B9] Hickey RM Ross RP Hill C Controlled autolysis and enzyme release in a recombinant lactococcal strain expressing the metalloendopeptidase enterolysin A Appl Environ Microbiol 2004 70 3 1744 1748 10.1128/AEM.70.3.1744-1748.2004 15006800 PMC368307

[B10] Ruyter PGGAd Kuipers OP Meijer WC Vos WMd Food-grade controlled lysis of *Lactococcus lactis *for accelerated cheese ripening Nature Biotechnology 1997 15 10 976 979 10.1038/nbt1097-976 9335048

[B11] Baneyx F Recombinant protein expression in *Escherichia coli* Current Opinion in Biotechnology 1999 10 5 411 421 10.1016/S0958-1669(99)00003-8 10508629

[B12] Vincentelli R Bignon C Gruez A Canaan S Sulzenbacher G Tegoni M Campanacci V Cambillau C Medium-scale structural genomics: strategies for protein expression and crystallization ChemInform 2003 34 20 no no 10.1021/ar010130s 12641473

[B13] LaVallie ER McCoy JM Gene fusion expression systems in *Escherichia coli* Current Opinion in Biotechnology 1995 6 5 501 506 10.1016/0958-1669(95)80083-2 7579661

[B14] Smith DB Johnson KS Single-step purification of polypeptides expressed in *Escherichia coli *as fusions with glutathione S-transferase Gene 1988 67 1 31 40 10.1016/0378-1119(88)90005-4 3047011

[B15] di Guana C Lib P Riggsa PD Inouyeb H Vectors that facilitate the expression and purification of foreign peptides in *Escherichia coli *by fusion to maltose-binding protein Gene 1988 67 1 21 30 10.1016/0378-1119(88)90004-2 2843437

[B16] Nilsson B Abrahmsén L David VG Fusions to staphylococcal protein A Methods in Enzymology 1990 185 Academic Press 144 161 2199777 10.1016/0076-6879(90)85015-g

[B17] Davis GD Elisee C Newham DM Harrison RG New fusion protein systems designed to give soluble expression in *Escherichia coli* Biotechnology and Bioengineering 1999 65 4 382 388 10.1002/(SICI)1097-0290(19991120)65:4<382::AID-BIT2>3.0.CO;2-I 10506413

[B18] LaVallie ER Lu Z Diblasio-Smith EA Collins-Racie LA McCoy JM Jeremy Thorner SDEJNA Thioredoxin as a fusion partner for production of soluble recombinant proteins in *Escherichia coli* Methods in Enzymology 2000 326 Academic Press 322 340 11036651 10.1016/s0076-6879(00)26063-1

[B19] LaVallie ER DiBlasio EA Kovacic S Grant KL Schendel PF McCoy JM A thioredoxin gene fusion expression system that circumvents inclusion body formation in the *E. coli *cytoplasm Nature Biotechnology 1993 11 2 187 193 10.1038/nbt0293-187 7763371

[B20] Waugh DS Making the most of affinity tags Trends in Biotechnology 2005 23 6 316 320 10.1016/j.tibtech.2005.03.012 15922084

[B21] Esposito D Chatterjee DK Enhancement of soluble protein expression through the use of fusion tags Current Opinion in Biotechnology 2006 17 4 353 358 10.1016/j.copbio.2006.06.003 16781139

[B22] Hu Y-c Baculovirus as a highly efficient expression vector in insect and mammalian cells Acta Pharmacol Sin 2005 26 4 405 416 10.1111/j.1745-7254.2005.00078.x 15780188 PMC7091893

[B23] Daly R Hearn MTW Expression of heterologous proteins in *Pichia pastoris*: a useful experimental tool in protein engineering and production Journal of Molecular Recognition 2005 18 2 119 138 10.1002/jmr.687 15565717

[B24] Dickason RR Edwards RA Bryan J Huston DP Versatile *E. coli *thioredoxin specific monoclonal antibodies afford convenient analysis and purification of prokaryote expressed soluble fusion protein Journal of Immunological Methods 1995 185 2 237 244 10.1016/0022-1759(95)00119-U 7561134

[B25] Terzaghi BE Sandine WE Improved medium for lactic streptococci and their bacteriophages Appl Environ Microbiol 1975 29 6 807 813 10.1128/am.29.6.807-813.1975 PMC187084 16350018

[B26] Mc Grath S Neve H Seegers JF Eijlander R Vegge CS Brondsted L Heller KJ Fitzgerald GF Vogensen FK van Sinderen D Anatomy of a lactococcal phage tail J Bacteriol 2006 188 11 3972 3982 10.1128/JB.00024-06 16707689 PMC1482904

[B27] Maze A O'Connell-Motherway M Fitzgerald GF Deutscher J van Sinderen D Identification and characterization of a fructose phosphotransferase system in *Bifidobacterium breve *UCC2003 Appl Environ Microbiol 2007 73 2 545 553 10.1128/AEM.01496-06 17098914 PMC1796965

[B28] Holo H Nes IF High-frequency transformation, by electroporation, of *Lactococcus lactis *subsp. cremoris grown with glycine in osmotically stabilized media Appl Environ Microbiol 1989 55 12 3119 3123 16348073 10.1128/aem.55.12.3119-3123.1989 PMC203233

[B29] Seegers JFML Mc Grath S O'Connell-Motherway M Arendt EK van de Guchte M Creaven M Fitzgerald GF van Sinderen D Molecular and transcriptional analysis of the temperate lactococcal bacteriophage Tuc2009 Virology 2004 329 1 40 52 10.1016/j.virol.2004.07.003 15476873

[B30] O'Connell Motherway M Zomer A Leahy SC Reunanen J Bottacini F Claesson MJ O'Brien F Flynn K Casey PG Moreno Munoz JA Functional genome analysis of *Bifidobacterium breve *UCC2003 reveals type IVb tight adherence (Tad) pili as an essential and conserved host-colonization factor Proceedings of the National Academy of Sciences 108 27 11217 11222 10.1073/pnas.1105380108 PMC3131351 21690406

[B31] Bahey-El-Din M Griffin BT Gahan CG Nisin inducible production of listeriolysin O in *Lactococcus lactis *NZ9000 Microbial Cell Factories 2008 7 24 10.1186/1475-2859-7-24 18664263 PMC2515284

[B32] Sambrook J Fritsch EF Maniatis T Molecular cloning: a laboratory manual 1989 2 Cold Spring Harbor, NY: Cold Spring Harbor Laboratory Press

[B33] Towbin H Staehelin T Gordon J Electrophoretic transfer of proteins from polyacrylamide gels to nitrocellulose sheets: procedure and some applications Proc Natl Acad Sci USA 1979 76 9 4350 4354 10.1073/pnas.76.9.4350 388439 PMC411572

[B34] Stormo GD Schneider TD Gold LM Characterization of translational initiation sites in *E. coli* Nucleic Acids Research 1982 10 9 2971 2996 10.1093/nar/10.9.2971 7048258 PMC320669

[B35] LaVallie ER McCoy JM Smith DB Riggs P Enzymatic and chemical cleavage of fusion proteins 2001 John Wiley & Sons, Inc. 10.1002/0471142727.mb1604bs28 18265131

[B36] McCoy J LaVallie E Expression and purification of thioredoxin fusion proteins 2001 John Wiley & Sons, Inc. 10.1002/0471142727.mb1608s28 18265135

[B37] Lu Z DiBlasio-Smith EA Grant KL Warne NW LaVallie ER Collins-Racie LA Follettie MT Williamson MJ McCoy JM Histidine patch thioredoxins: mutant forms of thioredoxin with metal chelating affinity which provide for convenient purifications of thioredoxin fusion proteins Journal of Biological Chemistry 1996 271 9 5059 5065 10.1074/jbc.271.9.5059 8617783

[B38] Arai R Ueda H Kitayama A Kamiya N Nagamune T Design of the linkers which effectively separate domains of a bifunctional fusion protein Protein Engineering 2001 14 8 529 532 10.1093/protein/14.8.529 11579220

[B39] Siponen M Spinelli S Blangy S Moineau S Cambillau C Campanacci V Crystal structure of a chimeric receptor binding protein constructed from two lactococcal phages J Bacteriol 2009 191 10 3220 3225 10.1128/JB.01637-08 19286807 PMC2687176

[B40] Spinelli S Campanacci V Blangy S Moineau S Tegoni M Cambillau C Modular structure of the receptor binding proteins of *Lactococcus lactis *phages Journal of Biological Chemistry 2006 281 20 14256 14262 10.1074/jbc.M600666200 16549427

[B41] Sciara G Blangy S Siponen M Mc Grath S van Sinderen D Tegoni M Cambillau C Campanacci V A topological model of the baseplate of lactococcal phage Tuc2009 Journal of Biological Chemistry 2008 283 5 2716 2723 18045876 10.1074/jbc.M707533200

[B42] Vegge CS Brondsted L Neve H Mc Grath S van Sinderen D Vogensen FK Structural characterization and assembly of the distal tail structure of the temperate lactococcal bacteriophage TP901-1 J Bacteriol 2005 187 12 4187 4197 10.1128/JB.187.12.4187-4197.2005 15937180 PMC1151708

[B43] Vegge CS Vogensen FK Mc Grath S Neve H van Sinderen D Brondsted L Identification of the lower baseplate protein as the antireceptor of the temperate lactococcal bacteriophages TP901-1 and Tuc2009 J Bacteriol 2006 188 1 55 63 10.1128/JB.188.1.55-63.2006 16352821 PMC1317572

[B44] Serrano LM Molenaar D Wels M Teusink B Bron P de Vos W Smid E Thioredoxin reductase is a key factor in the oxidative stress response of *Lactobacillus plantarum *WCFS1 Microbial Cell Factories 2007 6 1 29 10.1186/1475-2859-6-29 17725816 PMC2174512

